# Development of fermentation and respiration bioprocesses for efficient nitrogen removal through microbial catabolism

**DOI:** 10.1016/j.engmic.2026.100293

**Published:** 2026-07-17

**Authors:** Ji Qi, Xiaoyan Ma, Jian Chen, Qiwei Guo, Fangang Meng

**Affiliations:** a School of Environmental Science and Engineering, Sun Yat-Sen University, Guangzhou, 510275, China; b Guangdong Provincial Key Laboratory of Environmental Pollution Control and Remediation Technology (Sun Yat-Sen University), Guangzhou, 510275, China

**Keywords:** Biological nitrogen removal, Fermentation, Volatile fatty acids, Microbial metabolism, Microbial community

## Abstract

Conventional activated sludge processes are primarily designed for nitrogen and phosphorus removal, with carbon transformation regarded as a concomitant process that supports downstream denitrification rather than a proactively regulated process. Inspired by the metabolic division of labor in gut ecosystems, we proposed and validated a metabolism-guided strategy for an experimental membrane bioreactor (MBR-E) with a prefermentation unit that couples upstream fermentation with downstream denitrification to restructure carbon flux toward more bioavailable electron donors for nitrogen removal. Long-term operation showed that MBR-E achieved significantly lower effluent total nitrogen (7.9 ± 2.4 mg/L) compared with the control MBR system (MBR-C, 12.3 ± 3.5 mg/L), which was attributed to its elevated specific denitrification rate. Influent organics were efficiently converted into volatile fatty acids (VFAs) via fermentation and shortening hydraulic retention time from 0.67 h to 0.5 h shifted VFA composition from propionate/butyrate dominance to acetate enrichment. Further, 16S rRNA gene sequencing demonstrated that functional denitrifiers and nitrifiers were selectively enriched in MBR-E. Co-occurrence network analysis revealed strengthened cooperative interactions and tighter functional coupling between carbon degradation and nitrogen removal in MBR-E. Overall, this study demonstrates that fermentation-driven carbon reprogramming can effectively regulate downstream respiratory pathways and reshape the microbial community structure, providing a novel approach for efficient nitrogen removal from low-carbon/nitrogen wastewater.

## Highlights

• Fermentation-respiration coupling strategy enhanced nitrogen removal efficiency.
• Shortened HRT drove carbon flux toward acetate dominance.
• Functional denitrifiers (*Dechloromonas, Thauera*) and *Nitrospira* were enriched.
• Carbon source regulation enhanced microbial cooperation and network connectors.
• Stochastic processes dominated sludge community assembly in the coupled system.

## Graphical abstract

**Figure:**
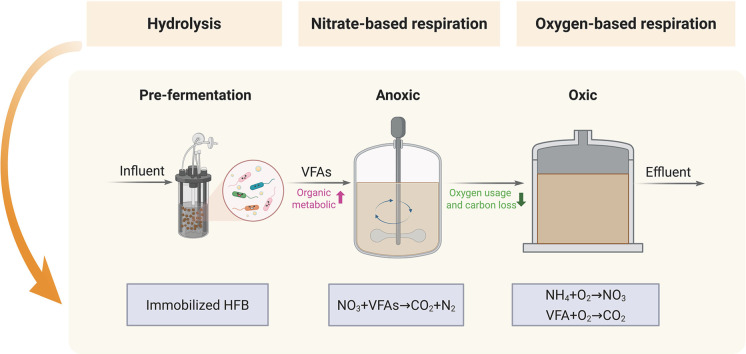

## 1 Introduction

Heterotrophic denitrification, widely employed for nitrogen pollution control due to its environmental friendliness, economic efficiency, and strong adaptability, remains a mainstream technology for nitrogen removal in biological wastewater treatment [1,2]. However, biological nitrogen removal performance is often constrained by the insufficient or delayed supply of bioavailable electron donors for denitrifiers, leading to the accumulation of intermediates (e.g., NO₂⁻, N₂O) and incomplete denitrification [3,4]. These limitations highlight the necessity of developing targeted strategies to optimize electron donor supply and carbon flux regulation in biological nitrogen removal systems.

The conventional anoxic-oxic (A/O) process is designed mainly based on nitrogen transformation routes: ammonia oxidation occurs in the oxic compartment, whereas denitrification occurs in the anoxic or anaerobic compartment [5]. Organic matter in sewage is an essential basal substrate for biological nutrient removal, providing the energy required for microbial metabolism and serving as a carbon skeleton for cell growth [6]. Therefore, to achieve complete nutrient removal, organic matter should be efficiently utilized in anoxic processes to drive nitrate reduction. Otherwise, residual biodegradable organics flowing into the oxic tank will compete with ammonium for dissolved oxygen, resulting in increased aeration consumption and the loss of valuable carbon sources. Notably, denitrifiers in biological systems are naturally accompanied by fermentative bacteria that convert complex and recalcitrant macromolecular organics into readily bioavailable volatile fatty acids (VFAs), which are the preferred high-quality electron donors for denitrifiers [7,8]. Thus, it is necessary to achieve the proactive conversion and targeted delivery of organic carbon.

Microbial redox reactions drive global biogeochemical cycles and serve as the core driving force of engineered biotechnologies for wastewater treatment [6,9]. In nature, heterotrophic fermentation and respiration function synergistically, with fermenters converting complex organics into reduced metabolites and electron carriers such as NADH and VFAs, which are then oxidized by respirers using external electron acceptors such as nitrate and oxygen [10,11]. The upstream supply of fermentation-derived VFAs and reduced cofactors can stabilize the tricarboxylic acid cycle and maintain the intracellular redox balance in denitrifiers, thus facilitating nitrogen removal [[12], [13], [14], [15]]. Furthermore, carbon source optimization for denitrification enhancement has revealed that small-molecule organics (e.g., acetate and methanol) deliver superior denitrification efficiency compared to macromolecular substrates (such as glucose and starch) because they impose a much lower metabolic burden on denitrifying bacteria than their macromolecular counterparts [[16], [17], [18]]. However, a major challenge for fermentation is that, although fermentative bacteria are ubiquitous in natural and engineered systems, they require extremely low oxidation-reduction potential (ORP) and tend to live in a free-living state, leading to severe biomass loss with effluent in continuous-flow treatment systems [19,20].

Gut ecosystems offer a natural analog for the metabolic division of labor, where fermentation-derived short-chain fatty acids are central intermediates shaped by microbial interactions and are subsequently utilized in downstream energy metabolism [21]. Inspired by this, herein, we focus on the microbial catabolism of influent organics and propose a novel fermentation-nitrate respiration-aerobic respiration coupled process for enhanced nitrogen removal. Such a design amplifies the coupling of microbial fermentation and organic matter respiration in engineered nitrogen removal. Specifically, this study aimed to: (1) investigate the conversion characteristics of influent organics to VFAs in a hydrolysis/fermentation bioreactor (HFB) and evaluate its nitrogen removal performance, (2) reveal the selective enrichment of denitrifying and acidogenic functional taxa, and (3) clarify the response of microbial community dynamics and assembly processes to HFB-mediated carbon source regulation. Overall, this study introduced a novel strategy for steering microbial division of labor and overcoming the carbon metabolism bottleneck observed during biological nitrogen removal.

## 2 Materials and methods

### 2.1 MBR set-up and operation

Two lab-scale anoxic/oxic membrane bioreactors (A/O MBRs) with identical configurations, each with an effective volume of 50 L, and anoxic and aerobic tanks of equal volume (25 L each) were operated continuously for more than 130 d (Fig. S1). The activated sludge collected from a municipal wastewater treatment plant (Guangzhou, China) was used as the inoculum. Three identical flat membrane modules (0.1 m^2^; pore size, 0.1 μm; PVDF; SINAP-10; Shanghai, China) were submerged in parallel within the aerobic tank. Synthetic municipal water with chemical oxygen demand (COD) of 300 mg/L, total nitrogen (TN) of 40 mg/L, and pH 7.23 ± 0.15 (see Table S1 for detailed composition) was fed directly into the anoxic tank of the control reactor (MBR-C).For the experimental reactor (hereinafter referred to as MBR-E), a compact HFB was installed upstream of the anoxic tank as a prefermentation unit to enrich the fermentative bacteria and avoid metabolic competition between fermentation and denitrification. The HFB tank, with an effective working volume of 0.96 L, was filled with high-density polyethylene carriers (K3, Jiangsu Yulong Environment Protection Co., Ltd, Jiangsu, China) at a filling ratio of 80% (a total of 150 pieces, each with a volume of 1 cm³). These porous carriers had a specific surface area of approximately 500 m²/m³ and a density of 0.94–0.97 g/cm³, providing attachment sites for fermentative bacteria and preventing biomass loss. The transmembrane pressure (TMP) was monitored using an online pressure sensor (Liance Automation, Hangzhou, China) to reveal fouling development. When the TMP increased to approximately 35 kPa, the fouled membrane modules were removed for physical and chemical cleaning treatments, immersing the membrane in 0.3% NaClO for 12 h, followed by treatment with 2.5% citric acid for 1 h.

The MBR operation was divided into three phases: start-up (0–14 days), stage I (15–60 days), and stage II (61–134 days). For MBR-C, a constant total hydraulic retention time (HRT) was maintained throughout the operation. For MBR-E, the HRT of the HFB tank was set to 0.67 h (start-up and stage I) and 0.5 h (stage II), with the HRT of the A/O unit being adjusted to 11.33 h for stage I and 11.5 h for stage II to maintain a constant total HRT of 12 h for the whole system. The constant flux in the MBR systems was set as 13.8 L/(m^2^·h) in this work (detailed operating conditions in Table S2). The effective volume of the HFB tank was specifically engineered to maintain a short HRT (0.5 and 0.67 h) under the target influent load, which is much lower than the 2–3 h typically reported for conventional fermentation processes. This configuration served as a metabolic regulation strategy to promote acidogenesis and was expected to limit methanogenic conversion [22], thus reducing potential VFA loss to methane and enhancing electron donor availability for downstream denitrification. The sludge retention time for the entire reactor was maintained at 20 d by discharging excess sludge (2.4 L/d) and the HFB consortia (0.1 L/d) regularly, which served to regulate biofilm turnover and prevent biomass overgrowth. Throughout the operation period, the mixed Liquor suspended solids (MLSS) concentration in the reactor was maintained at approximately 3500 mg volatile suspended solids (VSS)/L. Air diffusers were installed at the base of the membrane module to maintain an average dissolved oxygen (DO) concentration of 3.0 mg/L.

### 2.2 Characterization of the activated sludge and mass balance calculations

Batch tests were performed to assess the denitrifying activities of the sludge communities. The influent contained sodium acetate as the organic carbon source, with initial COD and NO₃⁻–N concentrations fixed at 350 mg/L and 35 mg/L, respectively, ensuring a sufficient carbon source for the denitrification process. Suspended solids (SS) and VSS, representing the biomass concentrations of the sludge, were determined following standard protocols [23]. The specific denitrification rate (SDNR) was determined based on a procedure described in a previous study [24]. Mass balance analyses of carbon were performed to evaluate the nutrient utilization efficiency during MBR operation (detailed calculations are shown in Table S3). Microbial viability was determined using a LIVE/DEAD BacLight Bacterial Viability Kit (Molecular Probes, Eugene, OR, USA) containing SYTO 9 and propidium iodide. Samples were stained in the dark at 25°C for 10 min, then mounted on glass slides and observed under a fluorescence microscope (MF31, Microvis Technology Co., Ltd., Guangzhou, China). The ratio of dead to live cells was quantified using ImageJ software.

### 2.3 DNA extraction, PCR amplification, and sequencing

During the operation of the MBRs, biomass samples were collected at the start-up phase on days 1–3 (S_1–S_3), from MBR-C sludge on days 7, 23, 46, 58, 70, 84, 98, 112, and 126 (C_7–C_126), from MBR-E sludge on days 7, 23, 46, 70, 86, 98, 112, 126, and 134 (E_7–E_134), and from the HFB tank on days 30, 60, 90, 120, and 134 (SJ_30–SJ_134), resulting in 21 sludge and 5 HFB samples for 16S rRNA gene sequencing. Genomic DNA was extracted from the collected biomass samples using a Fast DNA SPIN Extraction Kit (MP Biomedicals, Santa Ana, CA, USA). To amplify the V3-V4 hypervariable regions in the bacterial 16S rRNA gene, the primers 515F (5′-GTGCCAGCMGCCGCGGTAA-3′) and 907R (5′-CCGTCAATTCMTTTRAGTTT-3′) were employed. The purified amplicons were sequenced on an Illumina MiSeq platform (Biomarker Technologies Co., Ltd., Beijing, China). Raw sequencing data were analyzed using the BMK Cloud Platform (www.biocloud.com).

Calculation of alpha-diversity indices (including the ACE, Chao1, Shannon, and Pielou indices) and beta-diversity analyses was performed using Tutools (https://www.cloudtutu.com). Specifically, a beta-diversity analysis was performed based on the Bray–Curtis dissimilarity distance matrix at the amplicon sequence variant (ASV) level. Microbial interaction networks were constructed using the online Molecular Ecological Network Analysis platform (https://ieg2.ou.edu/MENA) based on the random matrix theory (RMT). Additional details regarding the construction of the network are available in a previous study [25]. Gephi software (version 0.10.1) was used for network visualization and modular analysis. Keystone taxa were recognized based on their respective topological positions, as specified by the values of within-module connectivity (Zi) and among-module connectivity (Pi) [26]. Additional details regarding the identification of keystone species are provided in Text S1.1.

### 2.4 Community assembly processes analysis

The Sloan neutral model (NCM), which quantitatively predicts the relationship between the occurrence frequencies of taxa and their relative abundance, was employed to determine the assembly mechanisms of the sludge communities [27]. In this model, according to the departure from the 95% confidence interval surrounding the neutral prediction, all communities were categorized into three projected partitions: above neutral, below neutral, or neutral. Moreover, phylogenetic bin-based null model (iCAMP) and beta-nearest taxon index (*β*NTI) analyses were performed to elucidate the relative importance of distinct ecological processes, utilizing the *R* packages “iCAMP” and “qpen” as well as scripts developed by Stegen et al. [28,29] (Text S1.2). Ecological processes were categorized as homogeneous selection (HoS), heterogeneous selection (HeS), dispersal limitation (DL), homogenizing dispersal (HD), and drift (DR).

### 2.5 Physicochemical analysis

The levels of MLSS, VSS, NH_4_^+^-N, NO_2_^–^-N, NO_3_^–^-N, total phosphorus(TP), TN, and COD were measured according to standard methods [23]. VFA concentrations were quantified using a gas chromatograph (GC-2014; Shimadzu, Japan) equipped with a flame ionization detector. The DO and pH of the mixed liquid in the reactor were monitored using DO (Oxi 3310, WTW, Germany) and pH meters (PHS-3D, Precision and Scientific Instrument Corp., Shanghai, China), respectively. The ORP of the sludge was measured using a portable detector (Rex YHBJ-262, Shanghai, China). EPS and SMP concentrations were measured according to the thermal extraction method [30]. The polysaccharide (PS) and protein (PN) contents of the SMP and EPS were measured according to the Dubois and Lowry methods, respectively [31,32].

### 2.6 Statistical analysis

All experimental data are presented as the mean ± standard deviation (SD). Independent-samples t-tests were used to evaluate significant differences between MBR-C and MBR-E. A value of *p* < 0.05 was considered statistically significant, and *p* < 0.01 was considered highly significant.

## 3 Results and discussion

### 3.1 Reactor performance

**Fig. 1:**
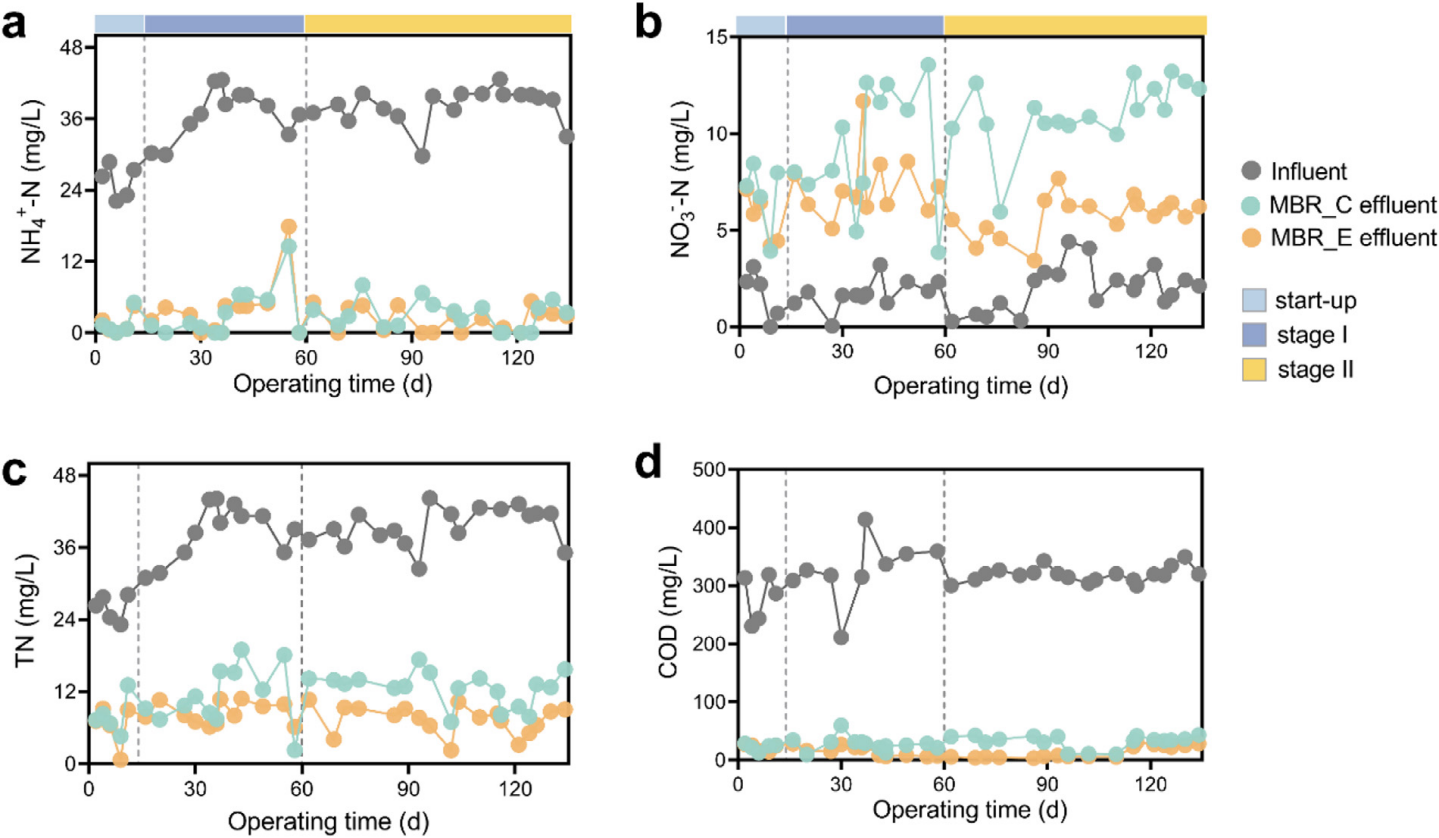
Organics and nutrient removal performance of MBR-C and MBR-E during the long-term operation. Profiles of (a) NH_4_^+^–N, (b) NO_3_^-^–N, (c) TN, and (d) COD concentrations in the influent and effluent. Operational stages: start-up (0–14 d), stage I (15–60 d), stage II (61–134 d), defined by the HRT adjustment of the HFB tank.

**Fig. 2:**
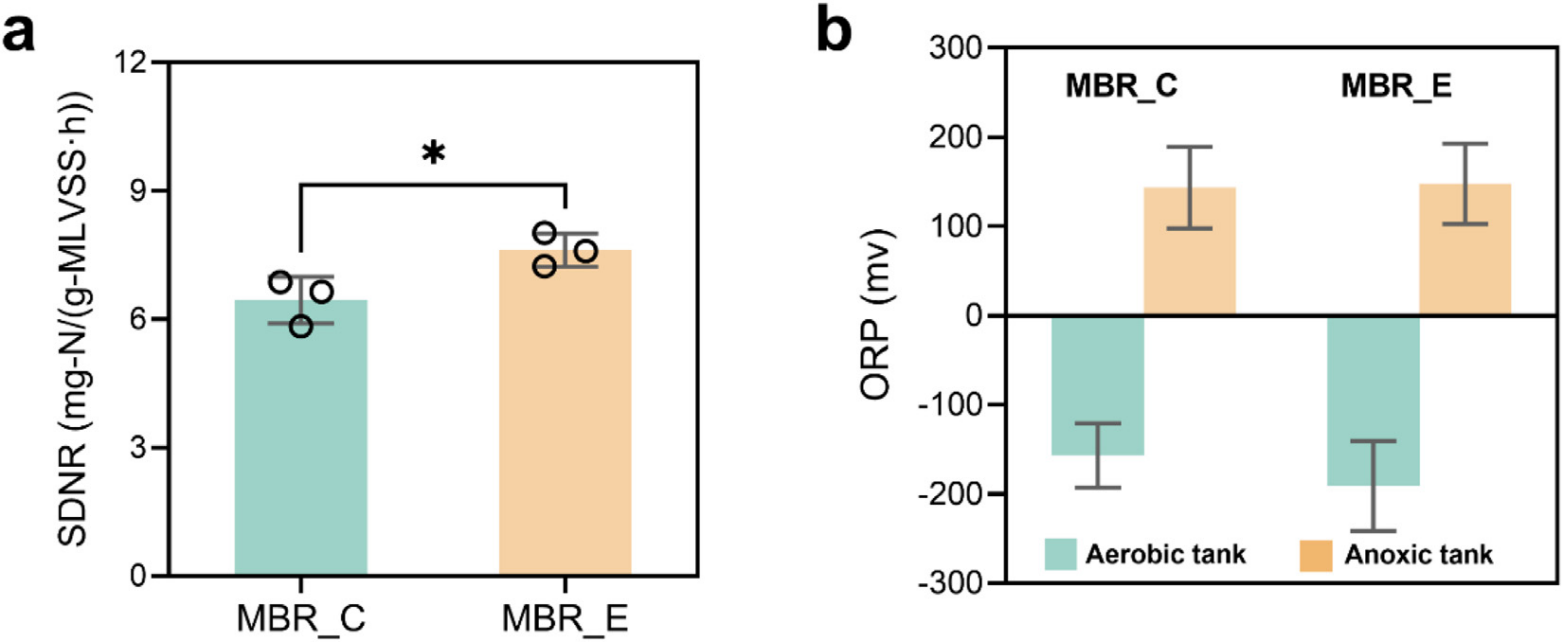
The activity of mixed liquor in stage II. (a) SDNR for the sludge from the MBR-C and MBR-E, respectively, (b) ORP values in aerobic tank, anoxic tank of the two MBRs. Symbols indicate the t-test results of multiple data sets at * indicates *p* < 0.05.

The performances of organics and nitrogen removal in the reactor were regularly monitored throughout the long-term operation over 130 d (Fig. 1, Table S4). During the whole operation period, under the same influent COD conditions, MBR-E achieved higher organic removal efficiency, with much lower permeate COD values (15.5 mg/L) than MBR-C (29.3 mg/L; *p* < 0.01). For nitrogen removal, the residual TN in the effluent was also reduced in MBR-E (7.9 ± 2.4 mg/L) compared with MBR-C (12.3 ± 3.5 mg/L; *p* < 0.01). Notably, the SDNR of the bulk sludge in MBR-E was elevated compared to MBR-C, increasing from 6.5 to 7.6 mg-N/(g-MLVSS⋅h) (Fig. 2A; *p* < 0.05).

Carbon mass balance analysis (Table S3) further showed that the proportion of the influent COD consumed during denitrification in MBR-E reached 28.1%, which was higher than that in the MBR-C (22.7%). This result demonstrates that the HFB-mediated prefermentation strategy achieved directional reconfiguration of the influent carbon flux, diverting a greater fraction of organic carbon toward nitrate reduction, thereby enhancing the overall nitrogen removal efficiency. Notably, the ORP values in the anoxic tank remained comparable between MBR-E (–168 mV) and MBR-C (–174 mV), indicating that the introduction of a prefermentation step did not compromise the reducing environment required for nitrate respiration (Fig. 2b). Overall, these results demonstrate that upstream fermentation improved the allocation and utilization of influent carbon for downstream denitrification, leading to the simultaneous enhancement of organic removal and nitrogen removal.

Additionally, MBR-E exhibited less membrane fouling than MBR-C, as evidenced by its TMP evolution profiles (Fig. S2). The average operation time for the TMP jump to reach ∼35 kPa was found to be 11 and 15 d for MBR-C and MBR-E, respectively, corresponding to fouling rates of 8.4 kPa/d and 6.0 kPa/d (Table S5). Meanwhile, the VSS concentration of the cake layer on the membrane surface was higher in MBR-C (25.8 ± 2.1 g/m²) than that of MBR-E (20.0 ± 1.5 g/m²). Furthermore, the corresponding total biopolymer concentration in the EPS of MBR-E (30.13 ± 15.31 mg/g-MLVSS) was significantly lower than in MBR-C (36.84 ± 7.52 mg/g-MLVSS; *p* < 0.01; Table S6).

### 3.2 Composition and dynamics of VFAs

**Fig. 3:**
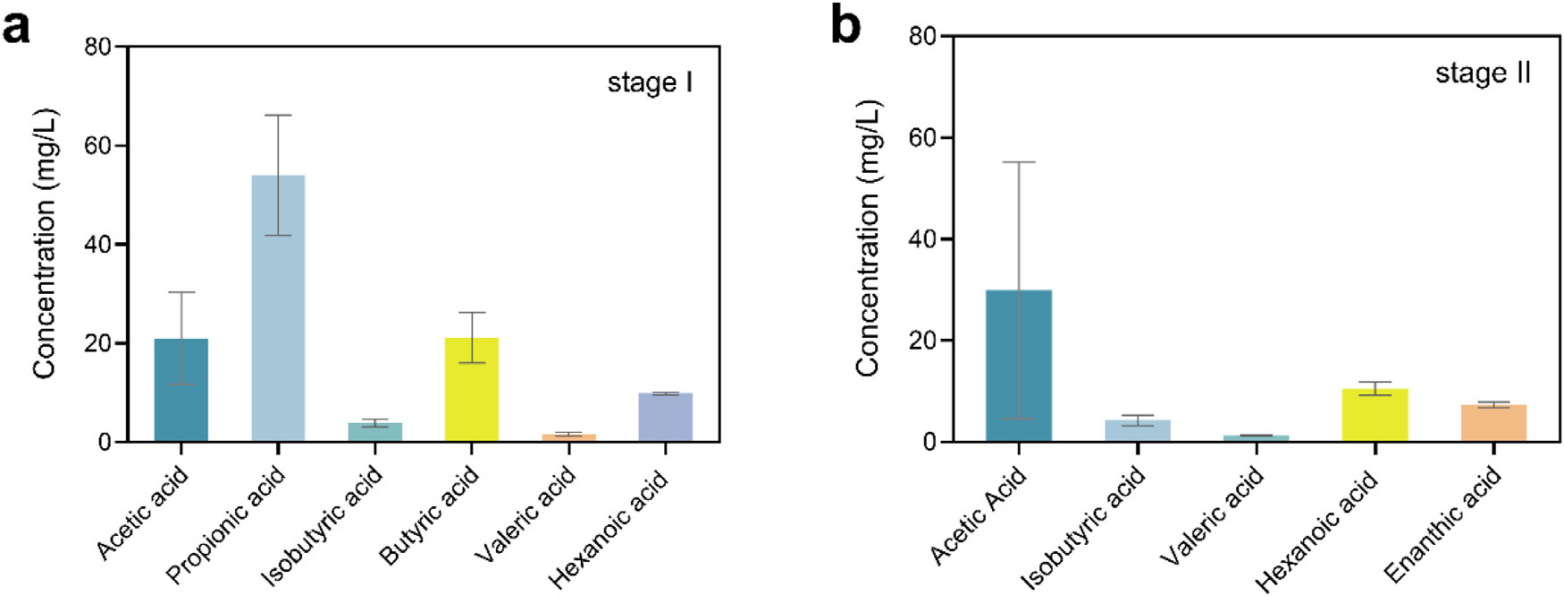
Dynamic changes of VFAs composition and yield under stable conditions in the HFB tank of MBR-E. Profiles of VFA compositions and concentrations in (a) stage I (15–60 d, HFB tank HRT = 0.67 h); (b) stage II (61–134 d, HFB tank HRT = 0.5 h).

To clarify the effect of prefermentation on carbon flux reformatting, the composition and carbon utilization efficiency of VFAs in the HFB tank effluent (integrated into MBR-E) were monitored during stable operation. During the entire operation, the continuous-flow HFB tank exhibited a distinct shift in VFA composition between the two time-based stages (Fig. 3, Table S7). In stage I, the effluent VFAs were dominated by propionic acid (54.0 ± 10.6 mg/L), followed by butyric (21.1 ± 4.4 mg/L), acetic (21.0 ± 8.6 mg/L), and hexanoic (9.8 ± 0.2 mg/L) acids. Trace amounts of isobutyric acid and valeric acid were also detected, collectively accounting for less than 5% of the total VFAs. In contrast, stage II exhibited a distinct VFA profile, with lower levels of propionic acid at 4.2 ± 1.0 mg/L and undetectable levels of isobutyric/butyric acids. Acetic acid levels in the fermentation broth reached 29.9 mg/L and emerged as the dominant VFA, with hexanoic acid remaining at a level similar to that in stage I.

This transition from a propionate/butyrate-dominated to an acetate-enriched VFA profile is directly driven by the ultrashort HRT regulation. Further shortening of the HRT substantially reduced the substrate residence time for fermentative microbes, which was insufficient to support the multistep enzymatic reactions and carbon chain elongation required for long-chain fatty acid synthesis [33]. Thus, acetate accumulation was selectively favored as the preferred high-quality electron donor for heterotrophic denitrifiers with the lowest metabolic burden. Although the total VFA concentration decreased, the bioavailable carbon supply for denitrification was enhanced (stage II). This redirection of carbon flux toward acetate is expected to benefit denitrifiers, given that acetate serves as a more efficient electron donor under carbon-limited conditions [34]. The shift in VFA composition was also tightly coupled with the succession of fermentative functional bacteria in the HFB, which is further discussed in the subsequent microbial community analysis.

### 3.3 Response of the microbial community structure

**Fig. 4:**
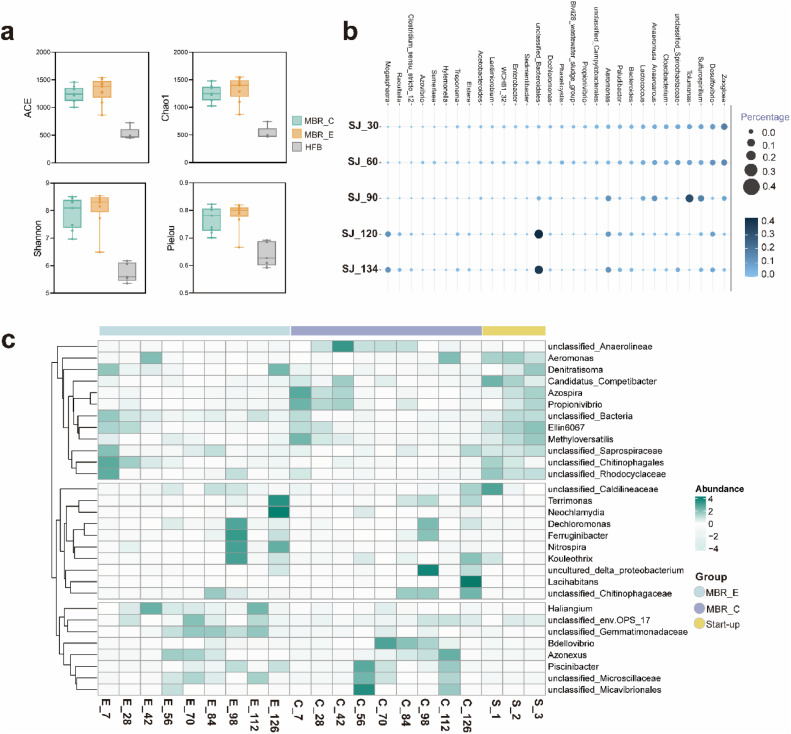
Microbial community diversity and composition of MBR-C sludge, MBR-E sludge and hydrolytic/fermentative consortia during the long-term operation. (a) *α*-diversity indices (i.e., ACE, Chao1, Shannon, and Pielou indices) of the sludge communities. Relative abundance of the top 30 genera from each group (b) HFB tank of MBR-E; (c) sludge communities of both reactors. For figure (b) and (c), the labels below (e.g., SJ_30, C_7, E_7, S_1, etc.) denote sampling time points, corresponding to the time intervals in the long-term operation. S_1–S_3 indicate start-up sludge sampled on days 1–3; C_7–C_126 indicate MBR-C sludge sampled on days 7, 23, 46, 58, 70, 84, 98, 112 and 126; E_7–E_134 indicate MBR-E sludge sampled on days 7, 23, 46, 70, 86, 98, 112, 126 and 134; and SJ_30–SJ_134 indicate HFB biomass sampled on days 30, 60, 90, 120 and 134.

As shown in Fig. 4A, there were clear trends in the biodiversity of the communities across different niches. The richness indices (ACE and Chao1) of the sludge communities from MBR-E were higher than those from MBR-C, whereas the HFB tank had the lowest species richness. The low Pielou index in the HFB tank indicated that the fermentative niche was dominated by a few specific functional taxa, consistent with the relatively low Shannon diversity observed in that setup. The reduced richness suggests that the short-HRT fermentative niche served as a specialized habitat that favored the enrichment of a limited number of acidogenic and syntrophic taxa. Beta-diversity analysis (principal component analysis) showed no statistically significant separation of the community structure between the two MBRs (Fig. S3), indicating that the overall community composition remained broadly similar. Therefore, the HFB-mediated regulation did not completely restructure the mainstream sludge community, but rather promoted niche-level functional enrichment and metabolic division of labor between upstream fermentation and downstream nitrogen removal.

Bacterial composition analysis revealed that the samples possessed similar dominant phyla (Fig. S4), with *Proteobacteria* (38.6%–50.7%) and *Bacteroidetes* (26.2%–44.7%) dominating the sludge and HFB communities. Other dominant phyla in the A/O units included *Chloroflexi* (5.9%–9.0 %), *Myxococcota* (5.6%–9.0%), and *Acidobacteriota* (4.1%–5.5%). The relative abundance of the phylum Chloroflexi was significantly higher in MBR-C sludge communities than in their MBR-E counterparts (9.0% *vs.* 5.9%, *p* < 0.01), indicating a stronger inhibition of filamentous bacteria (Fig. S5). Furthermore, the average relative abundance of *Nitrospirae* gradually increased from 1.5% to 2.5% in MBR-E, indicating that the segregated fermentation process effectively promoted the enrichment of nitrifying bacteria in the mixed liquor.

The top 30 genera were analyzed to reveal the mechanisms mediating denitrification (Fig. 4b–4c). In the HFB tank, early-stage communities were dominated by facultative fermenters and sulfate-respirers (e.g., *Lactococcus, Tolumonas*, and *Sulfurospirillum*), whose abundances gradually declined as VFAs accumulated [18,35,36]. In contrast, typical fermentative and syntrophic bacteria (e.g., *Aeromonas* and *Megasphaera*) are significantly enriched in the later stages of HFB operation [35,37]. Moreover, sludge community analysis showed that nitrogen-transforming bacteria such as *Dechloromonas* (5.6% *vs.* 4.9%), *Haliangium* (3.0% *vs.* 1.7%), *Nitrospira* (2.27% *vs.* 1.4%), and *Denitratisoma* (2.3% *vs.*1.0%) were more abundant in MBR-E sludge communities than those in MBR-C (respective abundances shown) [38]. The enrichment of denitrifiers was attributed to readily bioavailable VFAs supplied by upstream prefermentation, which reduced the metabolic burden of denitrifiers and enhanced nitrate reduction efficiency [[39], [40], [41], [42]]. Among them, the abundance of *Denitratisoma* initially declined, followed by continuous enrichment at 4.3% in MBR-E, as compared to the much lower value observed in MBR-C (1.0%, Fig. S6A). Notably, bacteria of the nitrifying genus *Nitrospira* were also significantly enriched in MBR-E, with their average relative abundance increasing gradually from 1.5% to 2.5% throughout stable operation (Fig. S6b), indicating the gradual enrichment of nitrifying capacity [43]. This trend was driven by carbon flux reconfiguration by the segregated HFB, in which VFAs were preferentially consumed for anoxic denitrification, thereby reducing the inflow of readily biodegradable COD into the aerobic zone and alleviating heterotrophic competition to favor slow-growing nitrifiers. Moreover, *Nitrospira* has been demonstrated to have robust acid tolerance, which enables it to effectively adapt to environments with slightly reduced pH caused by the inflow of fermentation broth (Fig. S7) [44,45].

Overall, the HFB-mediated fermentation-coupled system constructed a stable microbial functional network with a clear metabolic division of labor. The upstream HFB niche achieved directional enrichment of specialized fermentative bacteria for efficient VFA production whereas the mainstream MBR niche achieved selective enrichment of high-efficiency denitrifying and nitrifying bacteria for enhanced nitrogen removal.

### 3.4 Bacterial assembly of the sludge communities

**Fig. 5:**
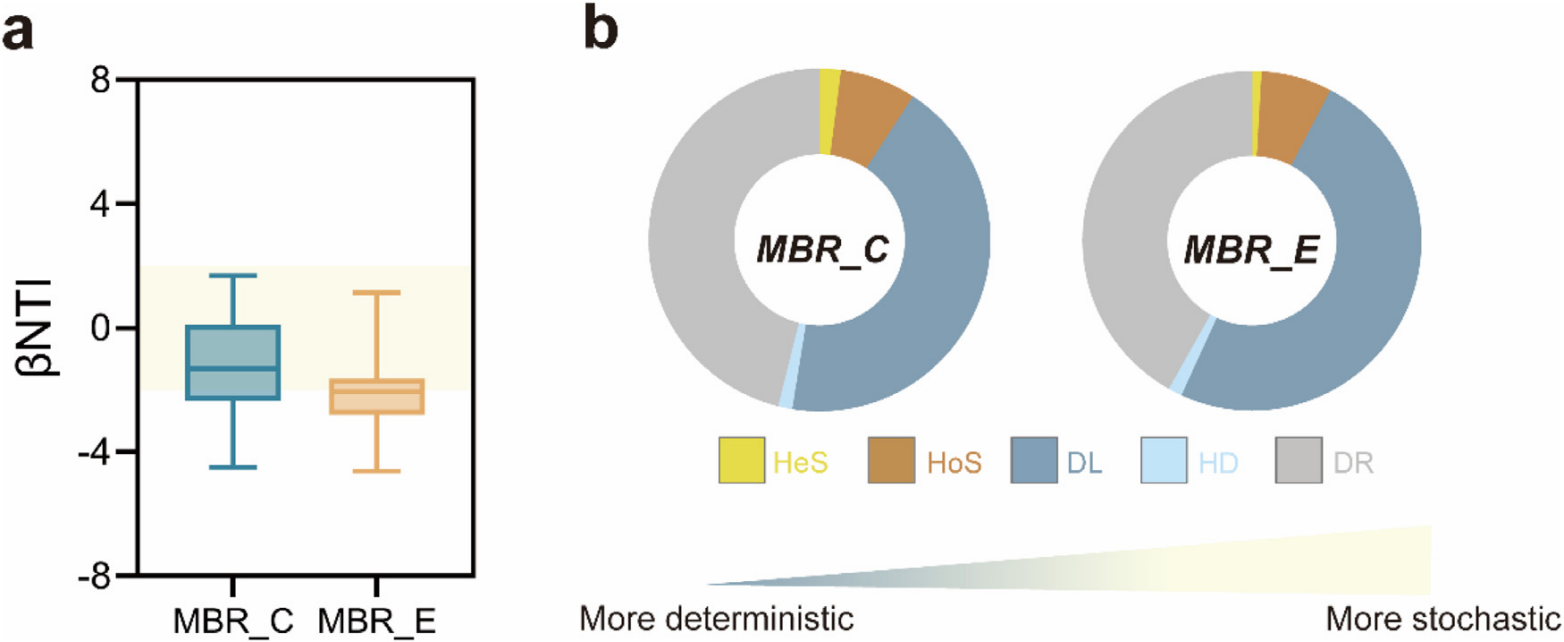
Bacterial assembly of MBR-C and MBR-E sludge communities. (a) *β*NTI values. (b) Relative importance of different assembly processes structuring the bacterial communities. Colors represent different assembly processes, i.e., heterogeneous selection (HeS), homogeneous selection (HoS), dispersal limitation (DL), homogenizing dispersal (HD), and drift (DR).

The NCM was used to quantify the contribution of the assembly-structured sludge communities (Fig. S8) [46]. For the NCM, the goodness-of-fit (R²) regarding sludge communities increased from 0.37 (MBR-C) to 0.4 (MBR-E), indicating a better fit to stochastic processes after hydrolytic/fermentative regulation. Furthermore, the migration rate (m) of the microbial community in MBR-E was significantly higher than that in MBR-C, indicating more frequent stochastic exchange and dispersal of microbial taxa across different functional niches. iCAMP analysis was performed to quantify the relative importance of different assembly processes (Fig. 5A). The mean *β*NTI values were negative (< 0) across samples but generally fell within the range of |*β*NTI| ≤ 2, indicating that strong deterministic selection was not the dominant force structuring the microbial communities. After HFB-mediated prefermentation regulation, the *β*NTI values of the MBR-E communities shifted toward –2 but still largely remained within the range |*β*NTI| ≤ 2, suggesting a weak tendency toward HoS in the fermentation-coupled system.

Consistently, HoS, DL, and DR dominated the bacterial community assembly, accounting for 5.9−7.2%, 35.8−49.5%, and 42.1−54.1%, respectively (Fig. 5b). Specifically, the relative contribution of HoS was lower in MBR-E than in MBR-C (5.9% *vs.* 7.2%), whereas DL played a more predominant role in structuring the communities in MBR-E (49.5% *vs*. 43.4% in MBR-C). In wastewater treatment systems, stochastic processes become more prominent when community turnover is strongly influenced by microbial migration and colonization, and functional redundancy among taxa [47,48]. Although the HFB-mediated pre-fermentation selectively favored specific metabolic functions (e.g., hydrolysis/fermentation and denitrification), these functions are phylogenetically widespread and often redundant across diverse microbial taxa. Meanwhile, the increased contribution of DL in MBR-E was likely associated with the spatial segregation created by the HFB–MBR configuration. The biofilm retained on K3 carriers physically immobilized core fermentative consortia. Such niche segregation likely restricted microbial exchange across compartments and therefore increased DL despite the targeted enrichment of functional microorganisms [49].

### 3.5 Bacterial community interactions

**Fig. 6:**
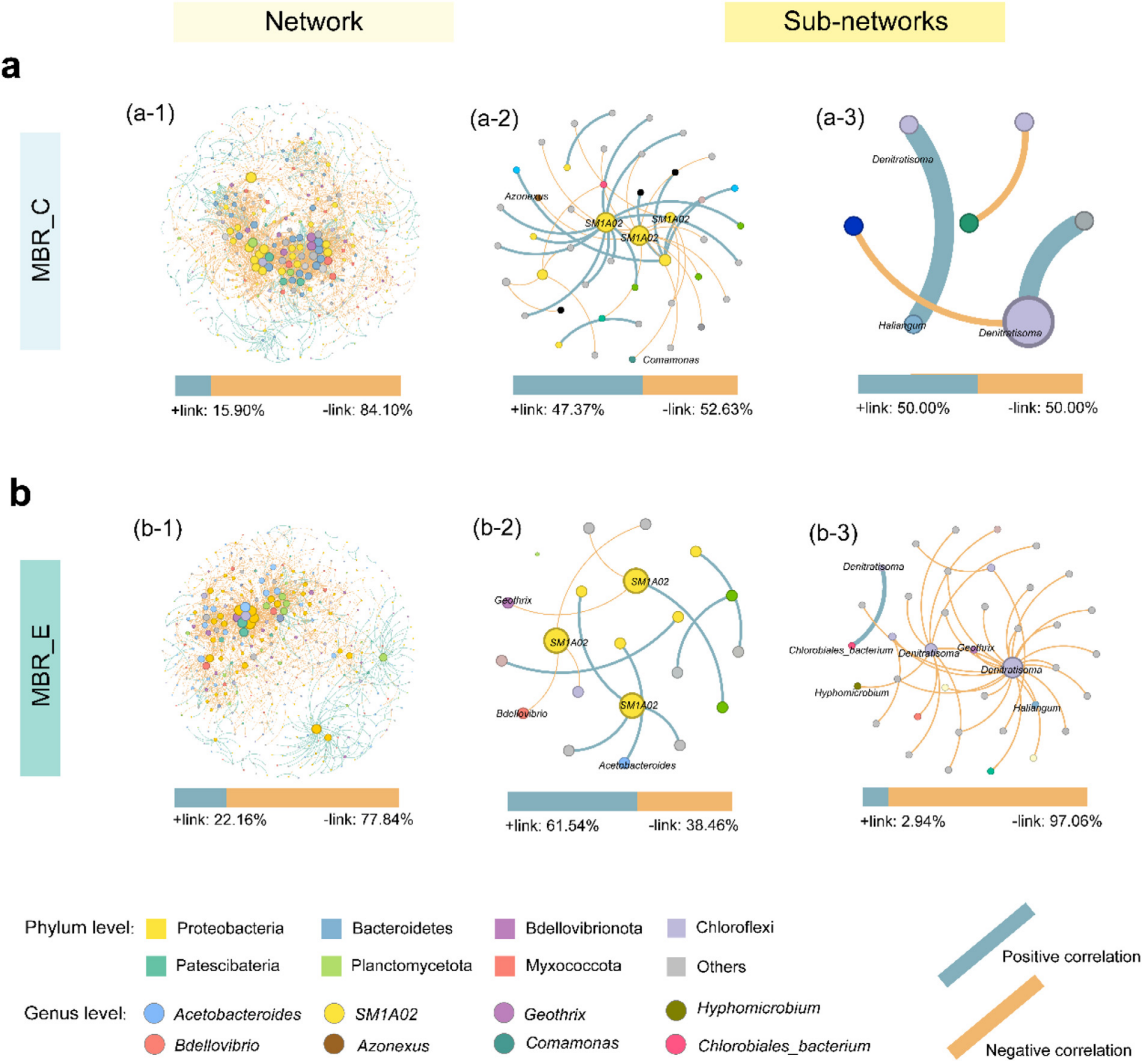
Phylogenetic molecular ecological networks (pMENs) constructed for microbial communities. (a-1) MBR-C sludge communities; (b-1) MBR-E sludge communities; (a-2) and (b-2) co-occurrence subnetwork of *SM1A02*; (a-3) and (b-3) co-occurrence subnetwork of *Denitratisoma.* The node colors denote the phylum of the ASVs. Connecting edges with light green and orange indicate positive and negative interactions, respectively.

Potential bacterial interactions were further revealed using RMT-based phylogenetic analyses. The constructed networks for both reactors exhibited scale-free characteristics, as indicated by *R²* values ranging from 0.7 to 0.8 (Table S8). The structural reorganization of microbial communities can influence the organizational principles of bacterial cells [50]. The MBR-E network had a shorter average path distance than MBR-C (3.4 *vs*. 4.4), suggesting a more interconnected architecture conducive to rapid metabolite and electron exchange between taxa [51]. Meanwhile, MBR-E exhibited a higher modularity index (0.59 *vs.* 0.56 in MBR-C) with fewer modules (19 *vs.* 31), suggesting clearer functional partitioning and stronger functional autonomy of the microbial community [52,53]. Notably, the MBR-C sludge community network contained 453 nodes and 2025 links (84.1% negative and 15.9% positive edges), whereas 468 nodes and 1530 links (77.8% negative and 22.2% positive edges) were observed in MBR-E counterpart (Fig. 6a-1, b-1). This result demonstrates that HFB-mediated prefermentation enhances cooperative interactions between dominant functional taxa.

Variations in network complexity further shape the ecological roles of individual components within a network [54]. The MBR-C sludge-associated network contained five module hubs and five connectors (Fig. S9 and Table S9), whereas the network constructed using the MBR-E sludge contained 10 module hubs and 15 connectors. Among the keystone taxa, *Ellin6067, SM1A02*, and *SBR1031* are associated with nitrification [55,56]. *SJA_28, Hyphomicrobium, Methyloversatilis*, and *Denitratisoma* are well-known denitrifying bacteria [38,57,58]. *Saprospiraceae* and *Chitinophagaceae*, which play critical roles in degrading complex refractory organic compounds and have been widely demonstrated to alleviate membrane biofouling, are also recognized as core keystone taxa.

To further explore the interspecific interactions of functional taxa, targeted subnetworks centered on the dominant functional microorganisms were constructed (Fig. 6a-2, b-2). In the subnetwork centered on *SM1A02*, MBR-E showed significantly more positive connections than MBR-C (61.5% *vs.* 47.4%). Notably, *SM1A02* exhibited a strong positive correlation with *Acetobacteroides* in MBR-E, suggesting a potential syntrophic cross-feeding relationship between the two genera. *Acetobacteroides* has been widely reported to ferment complex carbohydrates into acetate and hydrogen [59], which are critical metabolic intermediates that can be readily utilized by co-occurring denitrifiers as carbon sources and electron donors.

Moreover, the correlation between *Haliangium* and *Denitratisoma* was reversed from positive in MBR-C to negative in MBR-E (Fig. 6a-3, b-3), which is attributed to the altered availability and type of electron donors. In MBR-C, the influent organics were complex, had low bioavailability, and likely formed a synergistic relationship to support complementary denitrification metabolism under carbon limitation. However, in MBR-E, the HFB tank efficiently converted complex organics into readily utilizable VFAs, creating a shared resource pool that intensified niche competition between the two denitrifiers [60]. Notably, *Denitratisoma* in MBR-E formed a unique positive association with *Chlorobiales* bacteria, which mediates sulfide oxidation and generates reducing equivalents for sulfur metabolism and redox homeostasis [61]. This association suggests a potential metabolic coupling wherein *Chlorobiale*s may provide reducing power that supports denitrification.

### 3.6 Implication and limitations

In nature, the metabolic byproducts of one group frequently fuel another, forming efficient intrinsic fermentation-respiration couplings [62,63]. Based on this principle, we proposed a metabolism-guided strategy for wastewater treatment. This strategy regulates the downstream respiratory denitrification process by modulating upstream fermentative processes in a segregated HFB module to realize the targeted reconfiguration of carbon/electron donor speciation and the prevailing redox regime. Instead of bulk carbon addition, this strategy adjusts the carbon forms and redox states, which have been demonstrated to effectively drive the selective enrichment of functional microbes and optimize community assembly. Thus, this study highlights wastewater treatment systems as engineered microbial metabolic systems, in which targeted modulation of carbon and electron flows can support low-carbon nitrogen removal while maintaining microbial ecological structure and functional resilience [64,65]. However, some practical limitations still need to be considered, such as ensuring fermenter stability, matching fermentation output to downstream demand, and coping with dynamic influent fluctuations. Although the short HRT applied in the HFB demonstrated favorable performance in this study, maintaining stable fermentation under fluctuating influent conditions remains critical. Unstable fermentation may result in insufficient VFA production and subsequently disturb downstream nitrogen removal.

In practical municipal wastewater treatment, therefore, the fermentation output should be dynamically matched with the nitrate-reduction demand of the downstream A/O unit rather than being operated as a fixed pretreatment step. Full-scale robustness could be further improved through upstream flow equalization and adaptive operational strategies, such as real-time recirculation control or temporary bypass mechanisms. Besides, further investigations (e.g., transcriptomics and enzyme assays) could be conducted to reveal the inherent correlations between fermentation-derived carbon transformation and downstream nitrogen removal.

In addition, this study focused on the integration of nitrogen and carbon transformations without specifically considering phosphorus removal. The system adopted an A/O configuration without a dedicated anaerobic phosphorus-release zone, which did not provide favorable conditions for PAO metabolism and resulted in a limited TP removal efficiency (∼50%). The acetate-dominated VFAs generated by the HFB were preferentially consumed by denitrifiers for nitrate reduction, with a minor fraction utilized by aerobic heterotrophs for growth. VFAs were barely available for the PAOs because of the lack of anaerobic conditions, which further restricted the phosphorus removal performance of the current A/O configuration. Given that VFAs are key substrates for PAOs in enhanced biological phosphorus removal (EBPR) [66,67], the proposed fermentative strategy may has great potential for application in EBPR/DPAO-oriented configurations, where improved VFA availability and tailored carbon speciation can be fully exploited. Future work should therefore explore the integration of HFB-mediated carbon regulation with anaerobic–anoxic–oxic configurations for simultaneous carbon, nitrogen, and phosphorus removal.

## 4 Conclusion

This study proposed a metabolism-guided fermentation–respiration coupling strategy to enhance nitrogen removal from low C/N wastewater. By integrating an upstream HFB with a downstream A/O MBR, influent organic carbon was redirected toward more bioavailable electron donors, enabling MBR-E to achieve lower effluent TN and COD than the control system. The shortened HRT of the HFB promoted directional fermentation toward acetate-dominated VFAs, which provided readily available electron donors for heterotrophic denitrification. HFB-mediated carbon regulation further promoted the selective enrichment of functional denitrifiers, including *Dechloromonas* and *Haliangium*, and nitrifiers such as *Nitrospira*, while suppressing the filamentous fouling-related phylum *Chloroflexi*. In addition, fermentation coupling reshaped interspecific microbial interactions and community assembly processes, supporting functional stability and metabolic division of labor between upstream fermentation and downstream nitrogen removal. These findings demonstrate that upstream fermentation can regulate carbon speciation and improve carbon-efficient biological nitrogen removal.

## Data availability statement

All data analyzed during this study are included in this article and its supplementary information files. The raw sequencing datasets have been deposited in the NCBI Sequence Read Archive under accession numbers PRJNA1335936.

## CRediT authorship contribution statement

**Ji Qi:** Writing – original draft, Validation, Investigation. **Xiaoyan Ma:** Investigation, Formal analysis. **Jian Chen:** Investigation, Formal analysis. **Qiwei Guo:** Investigation, Formal analysis. **Fangang Meng:** Writing – review & editing, Supervision, Resources.

## Declaration of competing interest

The authors declare that they have no known competing financial interests or personal relationships that could have appeared to influence the work reported in this paper.

## Appendix Supplementary materials

Image, application 1
