# Supplementary material for: Development of fermentation and respiration bioprocesses for efficient nitrogen removal through microbial catabolism
Source: Eng Microbiol. 2026 Jul 17;6(3):100293. doi: 10.1016/j.engmic.2026.100293 (PMC13448423; doi:10.1016/j.engmic.2026.100293)
Supplement: Supplementary file 1 [file mmc1.docx]

***Supplementary Information for***

**Development of Fermentation and Respiration Bioprocesses for Efficient Nitrogen Removal Through Microbial Catabolism**

Ji Qi ^a,b^, Xiaoyan Ma ^a,b^, Jian Chen ^a,b^, Qiwei Guo ^a,b^, Fangang Meng ^a,b *^

^a^ School of Environmental Science and Engineering, Sun Yat-sen University, Guangzhou 510275, PR China

^b^ Guangdong Provincial Key Laboratory of Environmental Pollution Control and Remediation Technology (Sun Yat-sen University), Guangzhou 510275, PR China

*** Corresponding author**

Fangang Meng, Ph.D.

Email: [mengfg@mail.sysu.edu.cn](mailto:mengfg@mail.sysu.edu.cn)

Tel: 86-20-39335060

Fax: 86-20-84110267

Number of pages: 26

Number of figures: 9

Number of tables: 9

**Supplementary information**

1. **Supplementary Methods**

**1.1** Identification of keystone species

**1.2** Null model analysis

2. **Supplementary Tables**

**Table S1** Components and concentration of synthetic wastewater.

**Table S2** Operational parameters of MBR-C and MBR-E.

**Table S****3** Mass balance calculations for carbon based on the long-term monitoring in the MBRs.

**Table S4** Comparison of effluent quality between the control MBR and the experimental MBR.

**Table S5** Characteristics of the biocake formed at different fouling stages.

**Table S6** Variation of biopolymers in bulk sludge of the two MBRs

**Table S7** COD distribution of various VFAs of short-range HFB tank during operation.

**Table S8** Topological properties of the empirical pMENs of the microbial communities in MBR-C and MBR-E and their associated random pMENs.

**Table S9** Potential function of keystone species observed in ecological networks of microbial communities.

3. **Supplementary Figures**

**Figure S1** Schematic diagram of MBR-C and MBR-E treating synthetic municipal wastewater, respectively.

**Figure S2** A comparison of the fouling control between two MBRs.

**Figure S3** The profiles of beta-diversity of the bulk sludge microbiotas during the long-term operation. Both diversity analyses were conducted at the ASV level.

**Figure S4** Relative abundance of dominant microbial communities at phylum level.

**Figure S5** The microscopic visualization of the MBR-C and MBR-E bulk sludge.

**Figure S6** The relative abundance of the dominant genera (A) *Nitrospira*, (B) *Denitratisoma* at different sampling times in MBR-C and MBR-E.

**Figure S7** Profiles of pH values during long-term operation.

**Figure S8** Microbial community assembly of (A) MBR-C sludge, (B) MBR-E sludge.

**Figure S9** Z-P plot corresponding to the phylogenetic molecular ecological networks (pMENs) of ASVs in (A) MBR-C sludge, (B) MBR-E sludge.

**1. *Supplementary Methods***

**1.1 Identification of keystone species**

The topological role of each node could be determined by its within-module connectivity (Zi) and among-module connectivity (Pi) [1]. Nodes in each network were divided into four distinct categories: Peripherals (Zi ≤ 2.5 and Pi ≤ 0.62) exhibit limited connections within their module and rarely link to other modules; module hubs (Zi > 2.5 and Pi ≤ 0.62) maintain extensive connections with nodes within their module, playing a key role in sustaining the integrity of their respective module; connectors (Zi ≤ 2.5 and Pi > 0.62) serve to link multiple modules, thereby holding the network structure together and contributing to overall network integrity; and network hubs (Zi > 2.5 and Pi > 0.62) function as both module hubs and connectors, exerting critical effects on the coherence of both their own nodes and the entire network. Excluding peripherals, the remaining three categories are regarded as potential keystone taxa [2].

**1.2 Null model analysis**

Microbial community assembly was evaluated via null model analysis. The *β*-Nearest Taxon Index (*β*NRI) for pairwise comparisons among communities was used to assess how phylogenetic similarity deviates from stochastic expectations [3, 4]. The observed phylogenetic turnover was quantified using the abundance-weighted *β*-mean-nearest taxon distance (*β*MNTD) for each pairwise community comparison via the R package ‘picante’. *β*NTI represents the standardized effect size of observed *β*MNTD values in relation to the mean of the null distribution.

Pairwise comparisons with |*β*NTI| ≥ 2 are considered statistically significant, and the proportion of such comparisons is taken as a measure of selection's influence. Selection, one of the primary ecological processes shaping community structure, arises from fitness disparities among species, encompassing the effects of abiotic environmental filtering and biotic interactions. The fraction of pairwise comparisons with *β*NTI > +2 is interpreted as the impact of heterogeneous selection, which may result in phylogenetically dissimilar communities. In contrast, the proportion of pairwise comparisons with *β*NTI < -2 is taken as homogeneous selection, potentially leading to phylogenetically similar communities [5].

Additional analysis of comparisons with non-significant *β*NTI values (|*β*NTI| < 2) was performed via the Bray-Curtis-based Raup-Crick (RCbray) approach. |RCbray| ≤ 0.95 indicates the impact of ecological drift acting alone [3]. Moreover, RCbray > +0.95 and RCbray < -0.95 signify dispersal limitation and homogenizing dispersal, respectively.

**2. *Supplementary Tables***

**Table S1.** Components and concentration of synthetic wastewater.

|  | Substrates | Concentration (mg/L) | Trace elements | Concentration (mg/L) |
| --- | --- | --- | --- | --- |
| Ingredients | K_2_HPO_4_ | 17.56 | FeSO_4_⋅7H_2_O | 2.50 |
|  | NH_4_Cl | 152.75 | ZnCl_2_ | 0.06 |
|  | Glucose | 280 | MnCl_2_⋅4H_2_O | 0.06 |
|  | Peptone | 82 | Na_2_MoO_4_⋅2H_2_O | 0.19 |
|  | Beef extract | 28 | CoCl_2_⋅6H_2_O | 0.13 |
|  | NaHCO_3_ | 600 | NiCl_2_⋅6H_2_O | 0.04 |
|  |  |  | CuSO_4_ | 0.06 |
|  |  |  | H_3_BO_3_ | 0.06 |
|  |  |  | MgCl_2_⋅6H_2_O | 0.19 |
|  |  |  | CaCl_2_ | 0.44 |

**Table S2.** Mass balance calculations for carbon based on the long-term monitoring in the MBRs.

| **Parameter** | **MBR-C (Control)** | **MBR-E (Experimental)** |
| --- | --- | --- |
| Total HRT | 12 h (constant) | 12 h (constant) |
| HFB HRT | - | 0.67 h (Stage I), 0.5 h (Stage II) |
| A/O unit HRT | 12 h | 11.33 h (Stage I), 11.5 h (Stage II) |
| Flux | 13.8 L/(m²·h) | 13.8 L/(m²·h) |
| SRT | 20 d | 20 d |
| DO (aerobic tank) | 3.0 mg/L (average) | 3.0 mg/L (average) |
| MLSS (VSS) | ~3500 mg/L | ~3500 mg/L |
| HFB carrier | - | K3 carrier, 80% filling ratio |

**Table S3.** Mass balance calculations for carbon based on the long-term monitoring in the MBRs.

| Mass | Parameters | Calculations | control MBR (%) | Experimental MBR  (%) |
| --- | --- | --- | --- | --- |
| *COD* | *C_inf_* | *=C_eff_+C_exp_+C_P_+C_O_+C_DN_ =Q×S_COD-inf_* |  |  |
|  | *C_eff_* | *(1-q)×Q×S_COD-eff_* | 13.6 | 8.7 |
|  | *C_exp_* | *q×Q×X_V_×f_C_* | 27.4 | 28.4 |
|  | *C_P_* | *0.68^a^×Q×ΔS_TP_* | 0.4 | 0.4 |
|  | *C_O_* | *24×O_OUR_×V_Aer_-4.57^b^×Q×ΔS_NH4-N_* | 28.3 | 28.3 |
|  | *C_DN_* | *2.86^c^×N_DN_* | 22.7 | 28.1 |
| *COD balance rate* |  |  | 92.4 | 93.9 |

*a. 0.68: per gram phosphorus release requires 0.68gCOD;*

*b. 4.57:* *per gram NO_3_^-^-N production consumes 4.57g oxygen during the nitrification;*

*c.* 2.86: *per gram N_2_ generation consumes 2.86g COD during the denitrification* *(NO_3_^-^-N to N_2_).*

**Table S4.** Comparison of effluent quality between the control MBR and the experimental MBR.

| Parameter (mg/L) | Influent | Effluent | | *p*-value |
| --- | --- | --- | --- | --- |
|  |  | MBR-C | MBR-E |  |
| COD | 315±35.4 | 29.3±11.2 | 15.5±9.4 | <0.01 |
| TN | 37.2±8.1 | 12.3±3.5 | 7.9±2.4 | <0.01 |
| NO_3_^-^-N | 1.8±1.1 | 10.0±2.7 | 6.3±1.7 | <0.01 |
| TP | 4.8±1.4 | 2.5±0.8 | 2.4±0.9 | >0.05 |

**Table S5.** Characteristics of the biocake formed at different fouling stages.

| Items | Fouling stage | | Operating time | TMP (kPa) | Fouling rate (kPa/d) | VSS (g/m^2^) | Fouling resistance (m^-1^) |
| --- | --- | --- | --- | --- | --- | --- | --- |
| MBR-C | | Before TMP jump | 4.1 | 0 | 0.3 | - | - |
|  |  | After TMP jump ^a^ | 6.9 | 35 | 8.4 | 25.8 | 4.1×10^13^ |
| MBR-E | | Before TMP jump | 5.8 | 0 | 0.2 | - | - |
|  |  | After TMP jump ^a^ | 9.3 | 35 | 6.0 | 20 | 2.7×10^13^ |

a* The values listed in items are the average of 11 cycles of fouling from MBR-C and 9 cycles of fouling from MBR-E

**Table S6.** Variation of biopolymers in bulk sludge of the two MBRs

|  | Items | MBR-C | MBR-E |
| --- | --- | --- | --- |
| Biopolymers  (mg/g-MLVSS) | EPS-PS | 14.02±5.47 | 13.31±5.03 |
|  | EPS-PN | 22.71±3.94 | 19.02±7.62 |
|  | EPS | 36.84±7.52 | 30.13±15.31 |
|  | SMP-PS | 8.93±1.71 | 6.02±1.84 |
|  | SMP-PN | 8.19±3.12 | 8.75±3.03 |
|  | SMP | 17.12±4.03 | 14.77±3.44 |

**Table S7.** COD distribution of various VFAs of short-range HFB tank during operation.

| Types | Stage Ⅰ (HRT=0.67 h)\| | | Stage Ⅱ (HRT=0.5 h)\| |
| --- | --- | --- | --- |
|  | COD (mg/L) | COD (mg/L) | |
| Acetic Acid | 21.0±8.6 | 29.9±25.3 | |
| Propionic Acid | 54.0±10.6 | 4.2±1.0 | |
| Isobutyric acid | 3.9±10.6 | / | |
| Butyric acid | 21.1±4.4 | / | |
| Valeric acid | 1.6±0.4 | 1.3±0.1 | |
| Hexanoic acid | 9.8±0.2 | 10.8±1.4 | |
| Enanthic acid | / | 7.4±0.5 | |
| Total | 104.2±16.8 | 33.4±2.1 | |
| VFAs production efficiency (%) | 31.1±6.8 | 10.0±0.1 | |

*VFAs production efficiency was calculated as the ratio of total VFAs concentration to COD concentration [6]

**Table S8.** Topological properties of the empirical pMENs of the microbial communities in MBR-C and MBR-E and their associated random pMENs. *^a^*

|  |  | | **Empirical networks** | | | | | | | |  |
| --- | --- | --- | --- | --- | --- | --- | --- | --- | --- | --- | --- |
| Sample | network  size (*n*) | similarity  threshold  (st) | | *R*^2^ of power-law | average degree (avgK) | average path distance (GD) | harmonic geodesic distance (HD) | average clustering coefficient (avgCC) | modularity | transitivity  (trans) | Average  σ ^a^  (avgCC) |
| MBR-C | 453 | 0.93 | | 0.72 | 8.94 | 4.36 | 3.61 | 0.07 | 0.56 | 0.08 | 1.07 |
| MBR-E | 468 | 0.90 | | 0.77 | 6.54 | 3.44 | 4.15 | 0.07 | 0.59 | 0.06 | 0.20 |

*^a^* σ, small-word coefficient σ=(CC/CCr)/(HD/HDr), and σ > 1 represent “small-world” properties, indicating high interconnectivity and high efficiency [7]. Subscript r indicates the characteristics of the random network.

**Table S9.** Potential function of keystone species observed in ecological networks of microbial communities.

| Sample | ASV | Topological position | Lowest taxonomic rank | Affiliate Phylum | Functional description | Reference |
| --- | --- | --- | --- | --- | --- | --- |
| MBR-C  Sludge | ASV 18 | Module hubs | *o__Kapabacteriales* | Bacteroidota | Rarely described | - |
|  | ASV 56 | Module hubs | *o__WCHB1_41* | Verrucomicrobiota | Rarely described | - |
|  | ASV 107 | Module hubs | *f__Rhodanobacteraceae* | Proteobacteria | Rarely described | - |
|  | ASV 5185 | Module hubs | *f__**Diplorickettsiaceae* | Proteobacteria | Rarely described | - |
|  | ASV 2106 | Module hubs | *-* | - | - | - |
|  | ASV 478 | Connectors | *SC-I-84* | Proteobacteria | Nitrate-nitrogen removal | [8] |
|  | ASV 2108 | Connectors | *f_Vicinamibacteraceae* | Acidobacteriota | Denitrifier, resistant to metal | [9] |
|  | ASV 158 | Connectors | *c**_Anaerolineae* | Chloroflexi | Biofilm formation | [10] |
|  | ASV 739 | Connectors | *g__Flavobacterium* | Bacteroidota | Aerobic hydrocarbon degradation | [11, 12] |
|  | ASV 5082 | Connectors | *o__**Sphingobacteriales* | Bacteroidota | Produce glycosphingolipids | [13] |
|  | ASV 5288 | Network hub | *g__SM1A02* | Planctomycetota | Nitrification | [14] |
|  | ASV 19 | Module hubs | *g__Candidatus Competibacter* | Proteobacteria | Glycogen-accumulating organism  Utilize refractory organics | [15] [16] |
| MBR-E  Sludge | ASV 8508 | Module hubs | *g__**Bradyrhizobium* | Proteobacteria | Degrade aromatic  compounds | [17] |
|  | ASV 2077 | Module hubs | ***g__******Denitratisoma*** | Proteobacteria | Denitrification | [18] |
|  | ASV 232 | Module hubs | *g__**Candidatus Competibacter* | Proteobacteria | Glycogen-accumulating organism  Utilize refractory organics | [15] [16] |
|  | ASV 498 | Module hubs | *o__SBR1031* | Chloroflexi | Nitrification  Promote bacterial aggregation | [19, 20] |
|  | ASV 684 | Module hubs | *o__RBG_13_54_9* | Chloroflexi | Rarely described | - |
|  | ASV 6406 | Module hubs | *o__Sphingobacteriales* | Bacteroidota | Produce glycosphingolipids | [13] |
|  | ASV 8426 | Module hubs | *g__**Zoogloea* | Proteobacteria | Carbon removal, EPS production | [21] |
|  | ASV 5 | Module hubs | *g__**Methyloversatilis* | Proteobacteria | Heterotrophic denitrification | [22] |
|  | ASV 266 | Connectors | *-* | - | - | - |
|  | ASV 177 | Connectors | *g__**SM1A02* | Planctomycetota | Nitrification | [14] |
|  | ASV 5042 | Connectors | *g_**_**Hyphomicrobium* | Proteobacteria | Acetate-degrading denitrifier | [23] |
|  | ASV 266 | Connectors | *-* | - | - | - |
|  | ASV 195 | Connectors | *g*__*Ramlibacte*r | Proteobacteria | Rarely described | - |
|  | ASV 6423 | Connectors | *f__Verrucomicrobiaceae* | Verrucomicrobiota | Rarely described | - |
|  | ASV 480 | Connectors | ***g__******Ellin6067*** | Proteobacteria | Nitrification | [24] |
|  | ASV 200 | Connectors | ***g__Ellin6067*** | Proteobacteria | Nitrification | [24] |
|  | ASV 502 | Connectors | *f__**Chitinophagaceae* | Bacteroidota | Denitrification  Hydrolyzes SMP, BAP and EPS | [25] |
|  | ASV 2323 | Connectors | *g_**_**Azonexus* | Proteobacteria | N_2_ -fixing | [26] |
|  | ASV 518 | Connectors | *c__**Chitinivibrionia* | Fibrobacterota | Rarely described | - |
|  | ASV 2100 | Connectors | ***f__******Saprospiraceae*** | Bacteroidota | Breakdown of complex organic compounds | [27] |
|  | ASV 27483 | Connectors | ***f__Spirochaetaceae*** | Spirochaetota | Breakdown of complex organic compounds | [27] |
|  | ASV 3588 | Connectors | *o__**SJA_28* | Bacteroidota | Denitrification | [19] |
|  | ASV 152 | Connectors | *g__**Luteitalea* | Acidobacteriota | Rarely described | - |
|  | ASV 256 | Connectors | *-* | - | - | - |

**3. *Supplementary Figures***

**Figure S1**. Schematic diagram of MBR-C and MBR-E treating synthetic municipal wastewater, respectively.

**Figure S2.** A comparison of the fouling control between two MBRs. (A) Dynamic TMP profiles during long-term operation. (B) Biofilm formation potential of the bulk sludge from the MBR-C and MBR-E. Symbols indicate the t-test results: * *p* < 0.05, ** *p* < 0.01, *** *p* < 0.001, ns *p* > 0.05.

**Figure S3**. The profiles of beta-diversity of the bulk sludge microbiotas during the long-term operation. Both diversity analyses were conducted at the ASV level.

**Figure S4**. Relative abundance of dominant microbial communities at phylum level. Only the top 10 phyla are shown.

**Figure S5.** The microscopic visualization of the MBR-C and MBR-E bulk sludge. The pie charts of images at the bottom right based on the fluorescence images of live (green) and dead (red) cells.

**Figure S6.** The relative abundance of the dominant genera (a) *Denitratisoma*, (b) *Nitrospira* at different sampling times in MBR‒C and MBR‒E.

**Figure S7.** Profiles of pH values during long-term operation. CA refers to the anoxic tank of control MBR, CO refers to the aerobic tank of control MBR, EA refers to the anoxic tank of experimental MBR, EO refers to the aerobic tank of experimental MBR.

**Figure S8.** Microbial community assembly of (A) MBR-C sludge, (B) MBR-E sludge.  ASVs that occur more frequently than predicted by the model are shown in green while those that occur less frequently than predicted are shown in orange. Dashed lines denote 95% confidence intervals around the model prediction (pink line).

**Figure S9.** Z-P plot corresponding to the phylogenetic molecular ecological networks (pMENs) of ASVs in (a) MBR-C sludge, (b) MBR-E sludge. The topological feature of each node was determined based on the scatter patterns of Zi and Pi.

**References**

[1] J.M. Olesen, J. Bascompte, Y.L. Dupont, P. Jordano, The modularity of pollination networks, Proc. Natl. Acad. Sci. U.S.A. 104 (2007) 19891-19896.

[2] S. Banerjee, K. Schlaeppi, M.G. van der Heijden, Reply to ‘Can we predict microbial keystones?’, Nat. Rev. Microbiol. 17 (2019) 194-194.

[3] J.C. Stegen, X. Lin, J.K. Fredrickson, X. Chen, D.W. Kennedy, C.J. Murray, M.L. Rockhold, A. Konopka, Quantifying community assembly processes and identifying features that impose them, ISME J. 7 (2013) 2069-2079.

[4] C.J. Brislawn, E.B. Graham, K. Dana, P. Ihardt, S.J. Fansler, W.B. Chrisler, J.B. Cliff, J.C. Stegen, J.J. Moran, H.C. Bernstein, Forfeiting the priority effect: turnover defines biofilm community succession, ISME J. 13 (2019) 1865-1877.

[5] D. Ning, M. Yuan, L. Wu, Y. Zhang, X. Guo, X. Zhou, Y. Yang, A.P. Arkin, M.K. Firestone, J. Zhou, A quantitative framework reveals ecological drivers of grassland microbial community assembly in response to warming, Nat. Commun. 11 (2020) 4717.

[6] M. Atasoy, O. Eyice, A. Schnürer, Z. Cetecioglu, Volatile fatty acids production via mixed culture fermentation: Revealing the link between pH, inoculum type and bacterial composition, Bioresour. Technol. 292 (2019) 121889.

[7] Q.K. Telesford, K.E. Joyce, S. Hayasaka, J.H. Burdette, P.J. Laurienti, The ubiquity of small-world networks, (2011).

[8] Q. Sun, G. Zhu, Deciphering the effects of antibiotics on nitrogen removal and bacterial communities of autotrophic denitrification systems in a three-dimensional biofilm electrode reactor, Environ. Pollut. 315 (2022) 120476.

[9] Z. Chi, S. Ju, W. Wang, H. Li, Y.-H. Luo, B.E. Rittmann, Ethane-driven chromate and nitrate bioreductions in a membrane biofilm reactor, Chem. Eng. J. 452 (2023) 139135.

[10] C. Chen, Y. Yang, N.J.D. Graham, Z. Li, X. Yang, Z. Wang, N. Farhat, J.S. Vrouwenvelder, L.A. Hou, A comprehensive evaluation of the temporal and spatial fouling characteristics of RO membranes in a full-scale seawater desalination plant, Water Res. 249 (2024) 120914.

[11] B. Young, R. Delatolla, T. Abujamel, K. Kennedy, E. Laflamme, A. Stintzi, Rapid start-up of nitrifying MBBRs at low temperatures: nitrification, biofilm response and microbiome analysis, Bioprocess. Biosyst. Eng. 40 (2017) 731-739.

[12] J. Ribera-Pi, M. Badia-Fabregat, D. Arias, V. Gómez, E. Taberna, J. Sanz, X. Martínez-Lladó, I. Jubany, Coagulation-flocculation and moving bed biofilm reactor as pre-treatment for water recycling in the petrochemical industry, Sci. Total Environ. 715 (2020) 136800.

[13] A. Al Ashhab, M. Herzberg, O. Gillor, Biofouling of reverse-osmosis membranes during tertiary wastewater desalination: microbial community composition, Water Res. 50 (2014) 341-349.

[14] Q. He, Q. Zhang, J. Su, M. Li, B. Lin, N. Wu, H. Shen, J. Chen, Unraveling the mechanisms and responses of aniline-degrading biosystem to salinity stress in high temperature condition: Pollutants removal performance and microbial community, Chemosphere 362 (2024) 142688.

[15] K. Seguel Suazo, T. Dobbeleers, J. Dries, Bacterial community and filamentous population of industrial wastewater treatment plants in Belgium, Appl. Microbiol. Biotechnol. 108 (2024) 43.

[16] R.M. Castellanos, J.P. Bassin, D.M. Bila, M. Dezotti, Biodegradation of natural and synthetic endocrine-disrupting chemicals by aerobic granular sludge reactor: evaluating estrogenic activity and estrogens fate, Environ. Pollut. 274 (2021) 116551.

[17] A.C. Wong, E.A. Dinsdale, A. Pham, A.N. Pisarenko, R. Gersberg, M.E. Verbyla, Diversity and functional capabilities of the microbial metagenome in a potable water reuse system, Desalin. Water Treat. (2025) 101210.

[18] S. Cao, R. Du, B. Li, N. Ren, Y. Peng, High-throughput profiling of microbial community structures in an ANAMMOX-UASB reactor treating high-strength wastewater, Appl. Microbiol. Biotechnol. 100 (2016) 6457-6467.

[19] B. Su, Q. Liu, H. Liang, X. Zhou, Y. Zhang, G. Liu, Z. Qiao, Simultaneous partial nitrification, anammox, and denitrification in an upflow microaerobic membrane bioreactor treating middle concentration of ammonia nitrogen wastewater with low COD/TN ratio, Chemosphere 295 (2022) 133832.

[20] L. Zhang, G. Fu, Z. Zhang, Simultaneous nutrient and carbon removal and electricity generation in self-buffered biocathode microbial fuel cell for high-salinity mustard tuber wastewater treatment, Bioresour. Technol. 272 (2019) 105-113.

[21] J. Feng, X. Li, Y. Yang, X. Fan, Z. Zhou, J. Ren, X. Tan, H. Li, Insight into biofouling mechanism in biofiltration-facilitated gravity-driven membrane (GDM) system: Beneficial effects of pre-deposited adsorbents, J. Membr. Sci. 662 (2022).

[22] S. Zhai, M. Ji, Y. Zhao, X. Su, Shift of bacterial community and denitrification functional genes in biofilm electrode reactor in response to high salinity, Environ. Res.184 (2020) 109007.

[23] T. Lou, Z. Peng, K. Jiang, N. Niu, J. Wang, A. Liu, Nitrogen removal characteristics of biofilms in each area of a full-scale AAO oxidation ditch process, Chemosphere 302 (2022) 134871.

[24] Q. He, Q. Zhang, M. Li, J. He, B. Lin, N.P. Wu, J.J. Chen, X.H. Liu, X.Q. Dong, Harnessing diurnal dynamics: Understanding the influence of light-dark cycle on algal-bacterial symbiotic system under aniline stress, Bioresour Technol. 416 (2025) 131796.

[25] L. Zhong, C.Y. Lai, L.D. Shi, K.D. Wang, Y.J. Dai, Y.W. Liu, F. Ma, B.E. Rittmann, P. Zheng, H.P. Zhao, Nitrate effects on chromate reduction in a methane-based biofilm, Water Res. 115 (2017) 130.

[26] X. Hu, F. M. Kerckhof, J. Ghesquiere, K. Bernaerts, P. Boeckx, P. Clauwaert, N. Boon, Microbial protein out of thin air: fixation of nitrogen gas by an autotrophic hydrogen-oxidizing bacterial enrichment, Environ. Sci. Technol. 54 (2020) 3609-3617.

[27] S.J. McIlroy, P.H. Nielsen, The family saprospiraceae. Heidelberg: Springer (2014) p. 863–889.
